# A non-coding *CRHR2* SNP rs255105, a *cis*-eQTL for a downstream lincRNA AC005154.6, is associated with heroin addiction

**DOI:** 10.1371/journal.pone.0199951

**Published:** 2018-06-28

**Authors:** Orna Levran, Joel Correa da Rosa, Matthew Randesi, John Rotrosen, Miriam Adelson, Mary Jeanne Kreek

**Affiliations:** 1 The Laboratory of the Biology of Addictive Diseases, The Rockefeller University, New York, New York, United States of America; 2 Center for Clinical and Translational Science, The Rockefeller University, New York, New York, United States of America; 3 NYU School of Medicine, New York, New York, United States of America; 4 Dr. Miriam and Sheldon G. Adelson Clinic for Drug Abuse Treatment and Research, Las Vegas, Nevada, United States of America; Harvard Medical School, UNITED STATES

## Abstract

Dysregulation of the stress response is implicated in drug addiction; therefore, polymorphisms in stress-related genes may be involved in this disease. An analysis was performed to identify associations between variants in 11 stress-related genes, selected *a priori*, and heroin addiction. Two discovery samples of American subjects of European descent (EA, n = 601) and of African Americans (AA, n = 400) were analyzed separately. Ancestry was verified by principal component analysis. Final sets of 414 (EA) and 562 (AA) variants were analyzed after filtering of 846 high-quality variants. The main result was an association of a non-coding SNP rs255105 in the CRH (CRF) receptor 2 gene (*CRHR2*), in the discovery EA sample (*P*_*nominal*_ = .00006; OR = 2.1; 95% CI 1.4–3.1). The association signal remained significant after permutation-based multiple testing correction. The result was corroborated by an independent EA case sample (n = 364). Bioinformatics analysis revealed that SNP rs255105 is associated with the expression of a downstream long intergenic non-coding RNA (lincRNA) gene AC005154.6. AC005154.6 is highly expressed in the pituitary but its functions are unknown. LincRNAs have been previously associated with adaptive behavior, PTSD, and alcohol addiction. Further studies are warranted to corroborate the association results and to assess the potential relevance of this lincRNA to addiction and other stress-related disorders.

## Introduction

Opioids are highly addictive narcotics used mainly for pain management. Opioid addiction is a significant global public health challenge that is related in part to the unprecedented increase in the use of opioid pain relievers and more recently to the widespread availability of very inexpensive high-quality heroin and fentanyl [1]. Addictions are chronic relapsing diseases characterized by the compulsive use of a drug with destructive outcomes. Both genetic and environmental factors contribute to opioid addiction based on twin and family studies [2, 3]. Effective pharmacotherapies for opioid addiction include agonist maintenance with methadone and partial-agonist maintenance with buprenorphine as well as the antagonist treatment with extended-release injectable naltrexone.

Dysregulation of the stress response is a critical factor in the development of addiction [4]. There is a high inter-individual variability in the response to stress that is determined by an interaction of genetic and non-genetic factors. One of the mechanisms of the stress response is the adrenal secretion of glucocorticoids. Stress, endogenous opioids, and drugs of abuse modulate the hypothalamic-pituitary-adrenal (HPA) axis. Glucocorticoids regulate the activity of the HPA axis through negative feedback. Corticotropin-releasing hormone/factor (CRH/CRF) mediates the HPA axis and also acts in extra-hypothalamic regions to stimulate the mesocorticolimbic dopamine system that mediates the rewarding effects associated with drug use [5].

In our previous studies of stress-related genes that included samples that overlap the samples used in the current study, we have reported associations of variants in *FKBP5*, *GAL*, *CRHR2*, and *AVPR1A* [6–9] with heroin addiction. *GAL* SNPs were associated with opioid addiction by another group [10].

The purpose of this study was to expand previous studies by analyzing a larger number of single nucleotide polymorphisms (SNPs) within specific stress-related genes. Although the samples were genotyped for variants across the whole genome, we have decided *a priori* to concentrate on limited regions of a relevant function related to addiction thereby reducing the multiple testing burden. The number of subjects used in this study is relatively small but they were recruited with comprehensive ascertainment and stringent inclusion criteria. In addition, the ‘case’ subjects were preferentially selected from the extreme range of the addiction phenotype to increase the power of the study.

## Materials and methods

The present study focuses on two discovery samples that are part of a multi-ancestry sample (n = 1811) that included subjects with multiple addictions. This sample is shared by the Laboratory of the Biology of Addictive Diseases from the Rockefeller University with the National Institute on Drug Abuse (NIDA) Genetics Consortium. The current study was limited to subjects with heroin addiction (‘cases’), as the major addiction. Some of the subjects were also addicted or abusing cocaine and/or alcohol.

The current study includes only subjects with predominantly European (EA) or African ancestry (AA) from the USA (n = 1001). Self-described Hispanics were not included. The EA discovery sample included 601 people that were divided into 459 cases and 142 controls. The AA sample included 400 subjects that were divided into 227 cases and 173 controls.

Subjects were recruited at the Rockefeller University or at specific opiate substitution programs (e.g., Manhattan Campus of VA NY Harbor Health Care System, Weill Medical College of Cornell University, and The Dr. Miriam and Sheldon G. Adelson Clinic for Drug Abuse Treatment and Research, in Las Vegas).

Ascertainment of cases and controls was made by personal interview, performed in a similar manner at the recruiting places, using several instruments: the Addiction Severity Index [11], KMSK [12] and Diagnostic and Statistical Manual of Mental Disorders, 4th Edition (DSM-IV). All cases had a diagnosis of opioid dependence based on lifetime DSM-IV criteria, had a history of at least one year of multiple daily uses of heroin, and were in methadone maintenance treatment in the time of recruitment. The eligibility criterion for the control group was no diagnosis of illicit drug abuse. Subjects with excessive drinking or cannabis use were excluded, as described [6]. Subjects with active DSM-IV axis I disorder were excluded from the study.

The study was approved by the Institutional Review Boards of the Rockefeller University (for Rockefeller University and the Las Vegas clinic) and the VA New York Harbor Healthcare System. All subjects signed informed consent for genetic studies and sharing DNA with NIDA.

### A second EA case sample

An independent EA sample (n = 364) was analyzed to corroborate the most significant result obtained in the discovery EA sample. This sample is part of a larger sample obtained from the NIDA Clinical Trials Network study (CTN-0051), a comparative effectiveness trial of 24 weeks of treatment with extended-release naltrexone versus buprenorphine-naloxone [13]. Subjects were recruited at community treatment programs affiliated with the CTN and had DSM-5 opioid use disorder. All sites obtained local Institutional Review Board approval and all participants signed informed consent for genetic studies. This sample was not genotyped with the Smokescreen^®^ array. European ancestry contributions were assessed by Structure analysis in the majority (60%) of the sample. Nineteen samples from individuals who self-reported to have European ancestry were excluded based on Structure estimated European ancestry contribution < 70%. The rest of the sample was included based on self-report.

### Genotyping

DNA was extracted from blood and quantified using standard methods. The original multi-ancestry sample was genotyped with the Smokescreen^®^ array [14] at RUCDR Infinite Biologics at The Rutgers University, as part of the NIDA collaborative project of opioid addiction. Smokescreen^®^ is a genome-wide custom genotyping array of biallelic SNPs and simple indels with addiction-related gene content. CEL files of the current study samples were analyzed with Axiom^™^ Analysis Suite 2.0.0.3.5 (Affymetrix, Santa Clara, CA).

The second replication EA cohort was genotyped using Taqman pre-designed assay C_2267604_10, according to the manufacturer’s protocol (Thermo Fisher Scientific, Waltham, MA, USA). The majority of the sample (60%) was also genotyped for ancestry informative markers (AIMs) with Illumina GoldenGate Custom Panel, as described [15].

### Duplicates and relatives

Familial relationships and duplications were assessed in the original cohort via pairwise Identity by Descent (IBD) analyses in PLINK. The filtered genome-wide SNP set was used to calculate PI_HAT values, which represent the proportion of genome IBD sharing [16]. All known familial relationships were consistent with estimated genome sharing. Duplicates (PI_HAT > 0.99) and relatives (PI_HAT > 0.2) were excluded.

### Principal component analysis (PCA)

PCA was carried out on the original multi-ancestry cohort with the filtered genome-wide SNP set to investigate population structure, using the R package SNPRelate [17]. Seven PCs explained most of the variability of this set and were used for exclusion of outliers and adjustment of the groups that were originally based on self-reported ancestry. Three PCs were included as covariates in the analyses.

### Structure analysis

Structure analysis of 155 AIMs was performed for the second replication EA sample. Each subject was anchored against 1051 samples from 51 worldwide populations, as described [18]. This sample was not genotyped with the Smokescreen^®^ array. The markers were genotyped using an Illumina GoldenGate Custom Panel, as described [15].

### Stress-related SNP set

The current analysis was limited *a priori* to 11 stress-related genes (Table 1). SNPolisher and *Ps_Classification* functions (Axiom^™^ Analysis Suite) were used to select the best performing probes based on quality control metrics (e.g., call rate, hemizygosity, frequency, and cluster separation). A total of 846 high-quality informative variants (included 30 simple indels) were selected for analysis from these genes (S1 Table). Cluster plots were visually evaluated for the SNPs with the most significant results.

**Table 1:** Genes and variants details.

Gene		No. of Variants	Analyzed in EA	Analyzed in AA
*AVP*	arginine vasopressin	17	8	6
*AVPR1A*	arginine vasopressin receptor 1A	105	44	59
*AVPR1B*	arginine vasopressin receptor 1B	87	36	54
*CRH*	corticotropin releasing hormone	19	4	13
*CRHBP*	corticotropin-releasing hormone binding protein	46	22	34
*CRHR1*	corticotropin-releasing hormone receptor 1	243	146	183
*CRHR2*	corticotropin-releasing hormone receptor 2	106	45	71
*FKBP5*	FK506-binding protein 51	15	11	13
*MC2R*	melanocortin 2 receptor	121	53	79
*OXT*	oxytocin	53	29	29
*POMC*	pro-opiomelanocortin	34	16	21
Total		846	414	562

### Statistical analysis

Pairwise linkage disequilibrium (LD) (D’ and r^2^) was estimated using Haploview 4.2. LD blocks were identified using the D’ confidence interval bound of 0.7–0.98 [19]. The single-SNP association analyses were conducted using PLINK 1.9 [20] by logistic regression, under dominant or recessive model assumptions. The following filters were used: a. Exact tests for deviation from Hardy-Weinberg equilibrium (HWE) with a threshold of *P* = 0.005; b. MAF < 0.05; c. missing genotype data (< 90%). A maximum test statistic was applied to account for the dominant and the recessive model tests, using Sumstat [21]. Correction for multiple testing was performed by permutation test (n = 100,000), for the dominant model that showed nominally significant results, using PLINK. Sex and the PCs 1–3 were included as covariates.

The SNPs set used for association analysis was further pruned based on pairwise LD estimates using PLINK (window size of 300 SNPs, advanced by 25 SNPs and an r^2^ threshold of 0.8) separately for EA and AA. The EA LD-pruned set of 214 SNPs was used for permutation analysis in EA, and the AA LD-pruned set of 445 SNPs was used for permutation analysis in AA.

Analysis of the second EA case sample for the most significant SNP identified in the analysis of the discovery EA sample was conducted by logistic regression under the dominant model either separately or by adding the replication sample to the discovery EA case sample.

## Results

**Fig 1:**
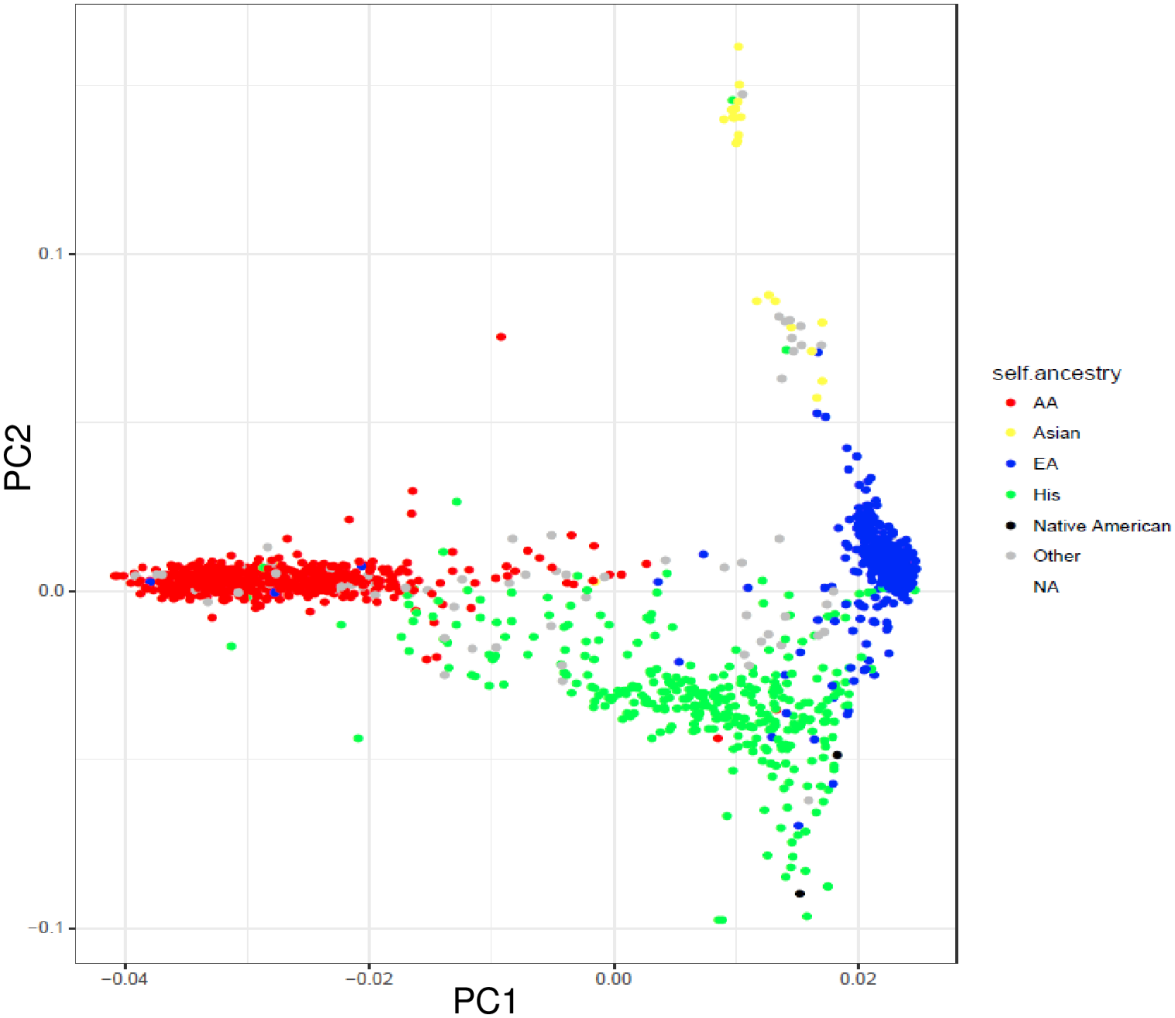
Scatter plot of the two main principal components based on filtered genome-wide genotype data of the original cohort. Each dot represents one individual colored by self-described ancestry. Color code: red: Africans; yellow: Asians; blue: Europeans; green: Hispanics; black: Native Americans; and gray: ‘others’. The corners correspond to high proportions of European, African, Asian and Native American ancestry.

Principal component analysis (PCA) was carried out on the original multi-ancestry cohort (n = 1811) using filtered genome-wide data from a custom addiction-related array Smokescreen^®^ [14] (Fig 1). The first PC distinguished between African and non-African ancestry. The second PC distinguished between European and Asian ancestry as well as between European and Native American ancestry. Hispanic and mixed samples showed intermediate values.

The two discovery samples (European ancestry and African ancestry) were defined based on data from seven PCs and analyzed separately. Outliers were excluded and misclassified samples were corrected. The three main PCs were included as continuous covariates in the analyses. Family members (PI_HAT > 0.2) were excluded based on the proportion of genome IBD sharing.

The current analysis was limited *a priori* to 11 stress-related genes (Table 1). A total of 846 high-quality informative variants (included 30 simple indels) were selected from this array (S1 Table).

### The European ancestry (EA) sample

The EA sample was divided into 459 cases (former heroin addicts in a methadone maintenance treatment program) and 142 controls. From the original set of 846 SNPs, 389 SNPs were excluded based on low frequency (minor allele frequency, MAF < 0.05), 40 additional variants were removed due to missing genotype data (< 90%), and three variants were removed due to Hardy-Weinberg disequilibrium (HWE). A final set of 414 variants (including eight simple indels) was used for the association analysis under two models of inheritance (dominant and recessive). Linkage disequilibrium (LD) analysis revealed that 110 of these SNPs can be tagged by 38 tag SNP (r^2^ = 1) (S1 Table). LD-pruned set (r^2^ < 0.8) of 200 SNPs was used for permutation analysis.

Comparison of genotype frequency distributions between cases and controls revealed significant differences in SNPs in three genes: *CRHR2*, *CRHR1*, and *FKBP5*. Table 2 lists the SNPs that passed the nominal threshold of *P* < 0.01 with sex and the three main PCs as covariates. The strongest association that remained significant after permutation analysis was detected for the non-coding *CRHR2* SNP rs255105 under the dominant model (*P*_*corrected*_ = 0.01).

**Table 2:** Top associations in the discovery EA sample (*P*_*nom*_ < 0.01).

Gene	SNP	Chr	Position^a^	Location	MAF^b^	Test	OR^c^	L95	U95	*P*_*nom*_	*P*_*perm*_
***CRHR2***	**rs255105**	**7**	**30692491**	**upstream**^d^	**0.26**	**D**	**2.1**	**1.4**	**3.11**	**0.00006**	**0.01**
	rs255112		30697517	upstream	0.28	D	1.7	1.1	2.42	0.00242	ns
	rs255107		30693607	upstream	0.32	D	1.7	1.2	2.53	0.00398	ns
	rs255108		30693639	upstream	0.34	D	1.7	1.1	2.47	0.00482	ns
	rs10271509		30692353	upstream	0.22	D	1.7	1.1	2.46	0.00934	ns
*FKBP5*	rs3800373	6	35574699	3' UTR	0.37	R	0.33	0.18	0.61	0.00056	0.052
	rs1360780		35639794	intron	0.39	R	0.36	0.20	0.63	0.00061	0.056
	rs6926133		35611598	intron	0.27	R	0.31	0.14	0.68	0.00291	ns
	rs4713899		35601504	intron	0.25	R	0.32	0.14	0.71	0.00423	ns
	rs9470080		35678658	intron	0.39	R	0.44	0.25	0.78	0.00619	ns
	rs4713916		35702206	intron	0.34	R	0.43	0.23	0.78	0.00648	ns
*CRHR1*	rs7225082	17	45771129	upstream	0.33	R	2.8	1.3	6.03	0.00646	ns

^a^ Assembly GRCh38.p7; Annotation Release 108

^b^ in controls

^c^ OR > 1 represents risk effect of the minor allele, OR < 1 represents a protective effect of the minor allele.

^d^ SNP rs255105 is located upstream of the main transcript and in an intron of transcript variant 2 and 3.

Box represents moderate to strong LD

Abbreviations: D: dominant; R: recessive; SNP: single nucleotide polymorphism; MAF: Minor allele frequency; OR: Odds ratio; L95: 95% confidence interval lower value; U95: 95% confidence interval upper value; *P*_*nom*_: nominal *P* value; *P*_*perm*_: *P* value of permutation test (n = 100,000) using the LD pruned set with three PCs and sex as covariates.

**Fig 2:**
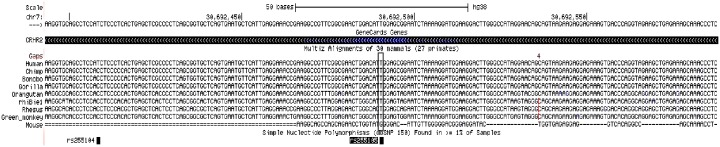
Evolutionary conservation of SNP rs255015 region. The ancestral T allele is highly conserved across representative species.

SNP rs255105 is highly conserved (Fig 2). The frequency of the ancestral T allele is ~30% in HapMap populations of European ancestry (26% in the EA control sample) and 90% in HapMap populations of African ancestry (83% in the AA control sample). The frequency of the carriers of the T allele (CT and TT) was 62% in the case sample compared to 43% in the control sample suggesting that the T allele is associated with increased risk for opioid addiction in this population (OR = 2.1).

### Independent EA case sample

The most significant result obtained in discovery EA sample (*CRHR2* SNP rs255105) was genotyped in an independent EA case sample (n = 364). The difference in genotype frequency distributions between the original controls sample (n = 142) and the second EA case sample remained significant in the same direction (*P* = 0.002). Analysis of the combined EA case samples (n = 823) was also significant (*P* = 0.0001; OR = 1.65; 95% CI 1.2–2.2).

### The African ancestry (AA) sample

The AA sample included 400 subjects that were divided into 227 cases and 173 controls. A total of 40 variants were removed due to missing genotype data (< 90%). A total of 239 SNPs were excluded based on low frequency (MAF < 0.05). Five variants were removed due to Hardy-Weinberg exact test. A final set of 562 variants was used for the association analysis under two models of inheritance (dominant and recessive). LD analysis revealed that 36 of these SNPs are in complete LD with another SNP in this set (S1 Table). LD-pruned set (r^2^ < 0.8) of 445 SNPs was used for permutation analysis.

Comparison of genotype frequency distributions between cases and controls revealed several SNPs with nominally significant differences but none of them survived permutation analysis. No association was detected for *CRHR2* SNP rs255105 in this sample. The T allele, the minor allele in the EA sample, is the major allele (83%) in the AA sample.

### Bioinformatics analysis

**Fig 3:**
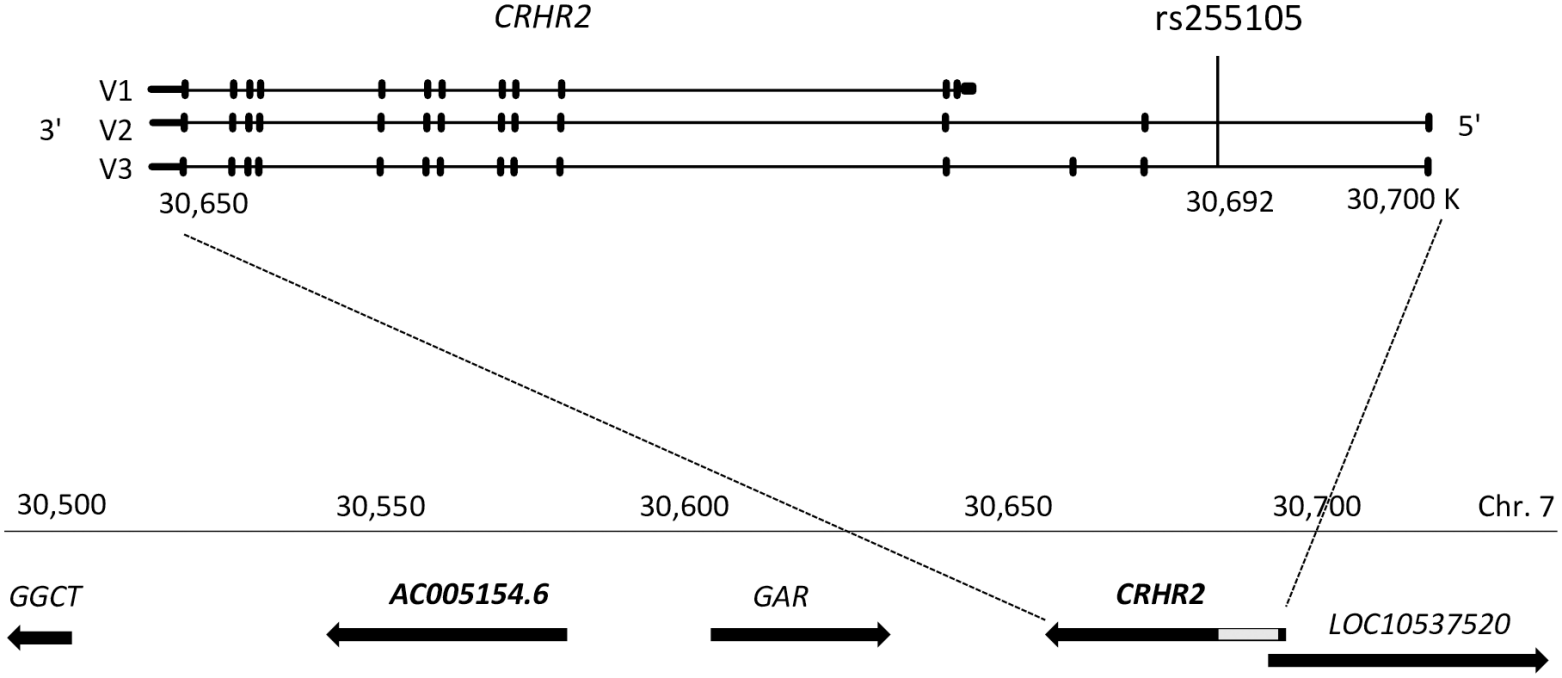
Genomic context of SNP *CRHR2* SNP rs255015. The genomic region spanning the *AC005154*.*6* and *CRHR2* genes. The main *CRHR2* transcripts (V1-V3) are shown.

The *CRHR2* (CRF2) gene (Chr 7:30,651,943–30,700,129, hg38) has several transcription variants that extend the 5' end of the gene [22]. SNP rs255105 is located upstream of the main transcript 1 (alpha, NM_001883.4) and in an intron of transcript variant 2 (beta, NM_001202475.1) and variant 3 (gamma, NM_001202481.1) (Fig 3). SNP rs255105 is also located upstream of the noncoding RNA LOC105375220 that overlaps transcript variants 2 and 3 in a reverse orientation (Fig 3).

**Fig 4:**
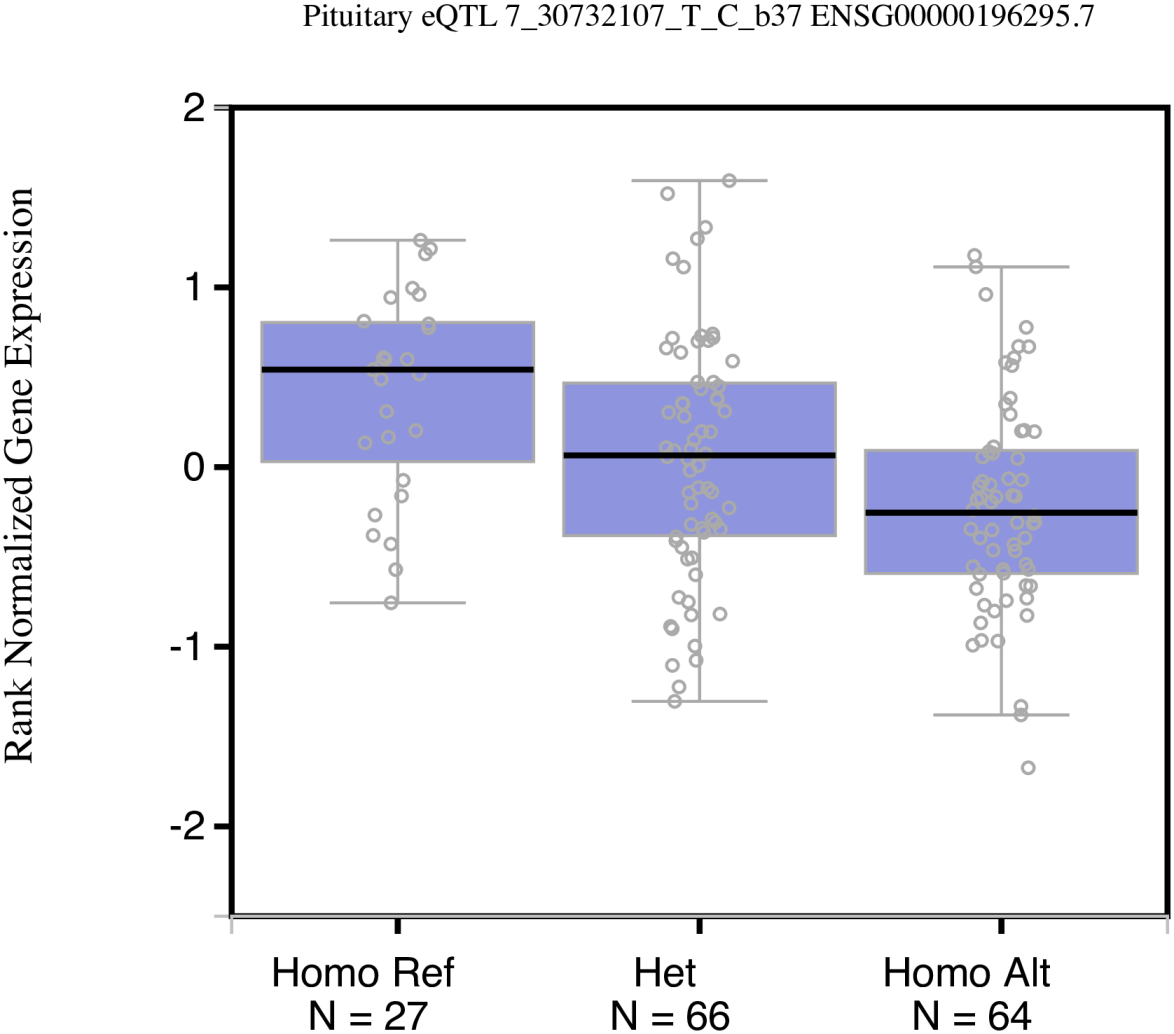
GTEx box plot showing relationships between SNP rs255015 (7_30732107_T_C_b37) genotype and lincRNA AC005154.6 (ENSG00000196295.7) expression in the pituitary (*P* = 0.000008). The sample groups of the different genotypes are indicated on the *X*-axis; and the relative expression level of lincRNA AC005154.6 is shown on the *Y*-axis. The T allele (the reference allele and the ancestral minor allele in the EA population) leads to higher expression of lincRNA AC005154.6. Homo Ref: homozygote for the reference T allele, Het: heterozygote; Homo Alt: homozygote for the alternative allele.

Data from the Genotype-Tissue Expression (GTEx) project release v6p [23] indicate that SNP rs255105 is a *cis*-expression quantitative trait locus (*cis*-eQTL) for a long intergenic non-coding RNA (lincRNA) gene *AC005154*.*6* (Chr 7: 30,516,309–30,594,809; hg38) (Fig 3). The ancestral T allele of rs255105 is associated with higher expression of this gene (*P* = 0.000008 in the pituitary) (Fig 4). Notably, there is inconsistency in the annotations of this lincRNA in different sources (AC005154.1, ENSG00000196295; LOC401320).

AC005154.6 is located downstream of the *CRHR2* gene between the genes *GGCT* (gamma-glutamylcyclotransferase) and *GARS* (glycyl-tRNA synthetase) (Fig 3). It has numerous alternative splice variants and its first exon overlaps the *GARS* gene first exon in the reverse orientation. The region overlapping *CRHR2*, *GARS*, *AC005154*.*6* and *GGCT* displays strong LD and several SNPs in this region including in *CRHR2* are associated with *AC005154*.*6* expression, but they are not in strong LD with rs255105.

**Fig 5:**
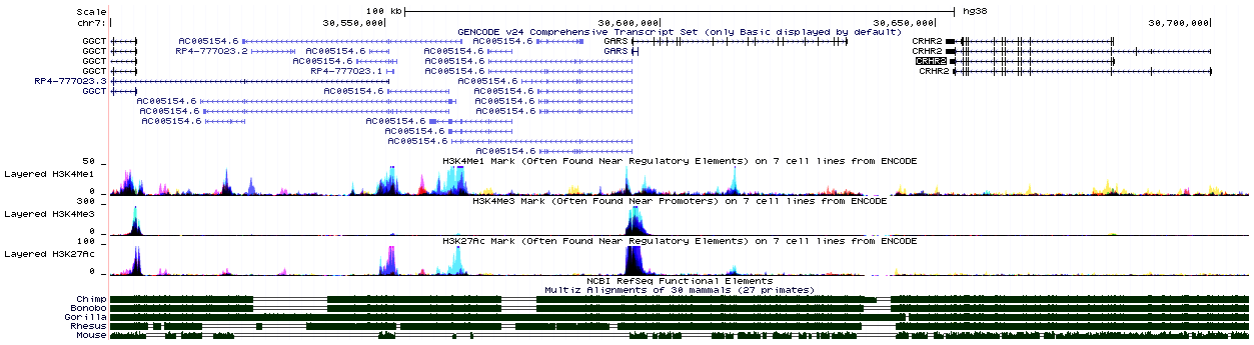
Multiple sequence alignment and histone marks of the genomic region of *AC005154*.*6* (USCS genome browser GRCh38/hg38; http://genome.ucsc.edu/).

Only a part of *AC005154*.*6* is evolutionally conserved among primates, and only small regions are conserved in rodents (Fig 5). There are several regions enriched with histone modifications (Fig 5). RNA-Seq data (GTEx) indicate expression of *AC005154*.*6* in various tissues with relatively higher expression in the pituitary.

## Discussion

The strongest association with opioid addiction in the current study was detected for the non-coding *CRHR2* SNP rs255105 in subjects with European ancestry. The result was corroborated when an independent EA sample was added to the analysis. The current study supplements our previous studies of stress-related genes in EA and AA [8, 9]. The previous EA study included a larger sample that included the majority of the current EA sample but was less homogenous and was limited in the number of SNPs analyzed per each gene. The main associations reported in the previous study were of *FKBP5* SNPs. The current study identified the same *FKBP5* associations although they did not reach significance after correction for multiple testing. Notably, the previous study also detected nominally significant association of *CRHR2* SNP rs255102 but did not detect an association with rs255105. In the current study, the association of SNP rs255102 did not reach nominal significance (*P* = 0.05).

SNP rs255105 is located upstream of the main *CRHR2* transcript and in an intron of several longer *CRHR2* transcript variants. Interestingly, it is significantly associated with expression of a downstream lincRNA gene AC005154.6 in several tissues. The ancestral T variant of SNP rs255105, associated with the risk of developing an opioid addiction, is highly conserved. The variant C allele is relatively rare (10%) in populations of African ancestry but more frequent (~30%) in populations of European ancestry. This change in allele frequency could indicate a positive selection toward the C allele that is suggested in this study to have a protective effect on the development of opioid addiction.

### CRH receptor 2

The CRH (CRF) family of peptides includes CRH and urocortin (UCN) 1–3. CRH is a neuropeptide released by the paraventricular nucleus of the hypothalamus following exposure to a stressor. It triggers the release of adrenocorticotropic hormone (ACTH) from the anterior pituitary, which in turn stimulates secretion of glucocorticoids from the adrenal gland [24]. Glucocorticoids activate the stress response and exert negative feedback on the HPA axis. CRH is also expressed as a neurotransmitter in extra-hypothalamic regions of the CNS.

CRH has two receptors: CRHR1 and CRHR2 that share ~70% of their amino acid sequences, but differ in their localization and binding affinities. They modulate anxiety-like behavior in a brain region- and cell-type dependent manner [25]. There is limited data on CRFR2 signaling but its effects seem to be plastic and dependent on past experience and ligand quantity. CRHR2 is highly expressed in the pituitary and is densely populated in brain structures involved in anxiety, fear, and arousal, such as the bed nucleus of the stria terminalis (BNST), a subregion of the extended amygdala that regulates the HPA axis. Rodent studies revealed an important role for CRHR2 in PTSD-like behavior [26], and suggest that optimal levels of CRHR2 are critical for coping with a traumatic event. Other rodent studies suggested that selective CRHR2 receptor agonists could be used as a therapy for nicotine addiction [27].

Several *CRHR2* SNPs have been reported to be associated with PTSD in EA women [28], and with major depressive disorder (MDD) in Japanese [29] but none of them is in LD with SNP rs255105 indicated in the current study. It remains to be determined if the identified SNP directly affects *CRHR2* expression or splicing.

### Long intergenic non-coding RNAs

The association of SNP rs255105 with the expression of a downstream lincRNA AC005154.6 is intriguing and may have clinical significance. Notably, lincRNA AC005154.6 is a *cis*-eQTL of other SNPs in the region including SNPs that are not in strong LD with SNP rs255105.

Our understanding of the complexity of the transcriptome has transformed as a result of improved RNA sequencing technologies. Non-coding RNAs (ncRNAs) constitute the majority of the transcriptome and are important transcriptional and posttranslational regulators [30, 31]. LncRNAs, a subclass of ncRNAs, are non-coding transcripts of >200 bp with exon-intron structure but no open reading frame. LncRNAs are transcribed in complex patterns relative to protein-coding genes and participate in processes like gene expression and epigenetic regulation through both cis- and trans-mediated mechanism. LincRNAs form RNA-protein complexes, modulate chromatin-regulatory proteins or transcription factors and affect the gene expression of nearby genes. They can also interact with miRNAs to modulate their expression and activity [32].

LncRNAs that are encoded between genes, like AC005154.6, are known as long intergenic non-coding RNAs (lincRNAs). LncRNAs play a role in a wide range of functions including adaptive behavior and neurological diseases and are potential new biomarkers and drug targets for stress-related disorders [33]. The majority of lncRNAs show tissue- and temporal-specific expression in the CNS. LncRNAs have been reported to be associated with more than 200 diseases including cancer, PTSD [34] depression [35], and schizophrenia [36].

The contribution of lncRNAs to the development of drug addiction is currently largely unknown and lncRNAs have not yet been characterized in animal models of addiction [37, 38]. Several lncRNAs were shown to be upregulated in the nucleus accumbens from human postmortem brain of heroin abusers compared to drug-free controls, using Affymetrix arrays [39]. Studies have identified lincRNAs that are involved in alcohol dependence [40]. Association studies revealed that alcohol dependence was associated with lncRNA LOC100507053 [41] and lncRNA LOC339975 [42].

The finding that only part of the AC005154.6 region is evolutionally conserved among primates and only small regions are conserved in rodents is important in the evaluation of its functionality. It also implies that the effects of this gene most likely cannot be studied in rodents and demonstrates that mice models may not be reliable as preclinical models for phenotypes caused by newly evolved lincRNAs.

### Conclusion

Hypothesis-driven study of variants in stress-related genes and heroin addiction identified an association with non-coding SNP in the *CRHR2* gene. A downstream lincRNA gene is a *cis*-eQTL of this SNP and additional SNPs in the region. This result is intriguing since lincRNAs play a role in adaptive behavior and neurological diseases and are potential drug targets for stress-related disorders. Further analyses are needed to corroborate the association result, to elucidate the potential biological mechanism underlying this lincRNA, and to assess the relevance of this lincRNA to heroin addiction and other stress-related disorders.

## Supporting information

S1 Table Selected variants details. (XLSX)
